# The influence of arts engagement on the mental health of isolated college students during the COVID-19 outbreak in China

**DOI:** 10.3389/fpubh.2022.1021642

**Published:** 2022-11-15

**Authors:** Yanying Chen, Xue Zeng, Lijian Tao, Junxiang Chen, Yuhui Wang

**Affiliations:** ^1^Department of Industrial Design, School of Architecture and Art, Central South University, Changsha, China; ^2^Department of Nephrology, Xiangya Hospital, Central South University, Changsha, China; ^3^Department of Nephrology, Hunan Key Laboratory of Kidney Disease and Blood Purification, The Second Xiangya Hospital, Central South University, Changsha, China; ^4^Xiangya Medical College, Central South University, Changsha, China

**Keywords:** COVID-19, arts engagement, anxiety, resilience, college students

## Abstract

**Objectives:**

The COVID-19 pandemic significantly impacted the mental health of college students. This study aimed to investigate the buffering effect of arts engagement on anxiety and resilience in college students during the COVID-19 pandemic.

**Study design:**

A cross-sectional study.

**Methods:**

The data were collected *via* an online survey during a wave of SARS-CoV-2 infections in Shanghai (March 15 to April 15, 2022). In total, 2,453 college students throughout China reported general anxiety symptom levels (according to the GAD-7), resilience (according to the Connor-Davidson Resilience Scale), frequency of receptive arts engagement in the previous year, exposure to risk situations, and behavioral changes due to the pandemic.

**Results:**

During the current stage of the pandemic, 43.7% of college students suffered from varying degrees of anxiety, and 2.6% showed severe anxiety. Gender and learning stage were not associated with anxiety. Hierarchical regression analysis showed that the decision to return to academic institution, the degree of exposure to COVID-19, and the frequency of accepting art participation and resilience could significantly predict the anxiety level of college students. Gender, study stage, behavioral changes arising from COVID-19, and exposure to COVID-19 significantly predict the resilience level of college students. There was an association between high frequency music activities, reading activities and low anxiety level (*p* < 0.001). There was an association between high frequency digital art, music activities, reading and high resilience (*p* < 0.01).

**Conclusions:**

Arts engagement appears to help students cope with mental health problems and those at risk. Policymakers should encourage college students to participate in art activities, especially in the context of social distancing.

## Introduction

According to the statistics on the official website of the National Health Commission of China, as of April 15, 2022, a total of 1,78,764 confirmed cases of COVID-19 have been reported nationwide. There were 25,956 confirmed cases (including 74 severe cases). A total of 28,25,543 close contacts (people who had a close contact with confirmed COVID-2019 patients) were traced, and 4,38,427 close contacts were under medical observation (1).Consequently, Chinese residents increasingly complied with recommended containment measures that are necessary under the time of crisis, but experienced high levels of anxiety, stress, and depression (2–4). For example, many colleges and universities in China closed, and their students suffered mental health issues (5–7). Xiong et al. (8) found that college students are most susceptible to mental health problems.

Arts engagement provides psychological, social, and behavioral benefits (9), advantages applicable to college students. Artistic and cultural participation can support cognitive reserve, enhance emotional regulation, and help prevent depression (10). Arts engagement can be categorized into receptive engagement (art that has been created and is now experienced by a listener, audience member, or gallery visitor) and participatory engagement (the creation of and participation in the arts) (11). Receptive arts engagement refers to listening to music, reading, visiting museums or watching movies, and has been demonstrated to be related to the reduction of depressive symptoms and anxiety symptoms (12, 13). Participatory arts engagement involves dancing, singing, drama performance and other activities. Research has proved that participatory arts engagement in these activities can improve the sense of experience happiness during COVID-19 (14). Arts engagement might serve as a coping mechanism in times of crisis.

Therefore, we analyzed the mental health status of college students during the COVID-19 pandemic. We also assessed predictors of anxiety and resilience among college students during the lockdown. Finally, we measured the relationship between each dimension of art participation and anxiety and resilience.

## Related work

### The psychological impact of COVID-19 on Chinese college students

Pandemics are associated with high stress levels due to fear of infection and death. To reduce the spread of disease and save lives, countries such as China promulgated public health measures, including closing academic institution and businesses. Segregation and pandemics significantly stressed the population (3, 6).

Although college students with COVID-19 may have a lower risk of severe complications, they face substantial difficulties after academic institution closures and social distancing.

The interruption of academic experience was a significant feature of the response to COVID-19. As many universities transition to distance learning, students face learning obstacles (15). Gillis et al. (16) found that most students reported distance learning-related disorders during the pandemic, leading to psychological problems such as anxiety. The pandemic also disrupted social relations, directly or indirectly moderating loneliness among college students. Palgi et al. (17) found that loneliness was a core risk factor for depression and anxiety during the COVID-19 pandemic. College students may also have many negative emotions (e.g., helplessness, depression).

These findings suggest that college students are susceptible to psychological problems during epidemics. Xiong et al. (8) found that college students are among the groups most susceptible to mental health problems. Compared with other groups, college students had more psychological problems. Li et al. (18) showed that the prevalence of depression and anxiety among college students during the COVID-19 pandemic were 39% and 36%, respectively. Zimmermann et al. (19) found that the prevalence and rate of depression and anxiety among college students were alarmingly high during the pandemic. Individuals with pre-existing psychological problems, females, and Latinos face a more significant risk of mental health problems. Haikalis et al. (20) showed that anxiety and depression symptoms among college students with COVID-19 increased.

Many studies identified several unhappy emotions among college students during the pandemic. College students as a vulnerable group should not be ignored during the COVID-19 pandemic. Some studies turned to focus on the coping strategies of College Students' negative emotions during the covid-19 pandemic (21–24). They put forward strategies such as developing distance education (21), strengthening physical exercise (22) and providing family support (23, 24). In our research, special attention is paid to the impact of art participation on the mental health of college students during the lockdown, with the objective to provide new perspectives for academic institution and policy supporters.

### Anxiety and resilience in COVID-19

Anxiety is a common psychological problem among college students during the pandemic (25, 26). Even under normal circumstances, students feel anxious. Among college students in Hong Kong (27), the prevalence of moderate anxiety was 12.2%, and severe anxiety was 5.8%. In Portugal, 15.6% of university students suffered moderate anxiety, while 8.3% suffered severe anxiety (28).

During the pandemic, college students are more likely to have psychological problems such as anxiety due to additional stress factors. Liu et al. (25) *f* found that public anxiety and depression levels rose during the pandemic. Wang et al. (29) found that Chinese college students showed higher anxiety about COVID-19 due to home isolation and other reasons.

Anxiety and depression have a high comorbidity rate (30). The two entities have overlapping symptoms, including fatigue, irritability, inattention, and sleep disorders. This finding suggests that anxiety and depression share a common psychopathological basis. Kessler et al. (31) found that 45.7% of patients with depression also suffer from anxiety disorders.

Resilience is defined as “the process of adapting well in the face of adversity, trauma, tragedy, threats, or significant sources of stress” (32). Resilience can promote the development of the individual and prevent negative emotions, thoughts, and behaviors.

Most people become stronger through resilience to cope with difficulties (33). Research on resilience showed that resilience is inversely associated with indicators of mental illness, including negative affect, depression, and anxiety. Resilience was positively correlated with mental health indicators, including positive impact, life satisfaction, subjective wellbeing, and prosperity (34). Especially during the COVID-19 pandemic, when college students experienced more stress than usual, resilience was expected to protect their wellbeing.

Resilience can reduce the adverse effects of stressors on mental health and promote positive mental health during difficult times like a pandemic. Ahorsu et al. (35) investigated resilience as a mediator of the relationship between positive, negative, and mental health during the pandemic. The authors found that resilience reduced the impact of negative influences and increased the impact of positive influences on mental health. Bozdag et al. (36) used an ecological framework to identify factors affecting the resilience of healthcare workers during the COVID-19 pandemic. These findings suggest that a better understanding of protective factors and related mechanisms of COVID-19 anxiety and resilience would be warranted to inform prevention and intervention efforts.

### Psychological effects of arts engagement

Artistic activities include imagination, sensory activation, cognitive stimulation, and social interaction. These components promote psychological, physical, social, and behavioral responses associated with managing mental health and wellbeing (10).

Research showed that arts engagement might benefit mental health (10).The role of art in emotional regulation also applies during the COVID-19 pandemic. Bu et al. (12) found that listening to music, reading, and engaging in artistic activities were associated with reduced depressive symptoms and anxiety and increased life satisfaction. Mak et al. (37) demonstrated that arts engagement improved the ability to cope with emotions during the lockdown. Drake et al. (11) found people engaging in artistic activities more often during than before the pandemic. Artistic activities (listening to music, reading and visual arts) regulated emotions most commonly by providing a means of escape.

Artistic activities can be divided into active and receptive (11). Active acts engagement refers to activities in which the individual was performing or making (e.g., playing a musical instrument, dancing, drawing). Receptive arts engagement refers to activities in which the individual was an audience member (e.g., listening to music, watching a dance concert, looking at visual art). These two kinds of artistic activities have been proven to improve people's wellbeing during the epidemic (13, 14).

Previous studies demonstrated a correlation between arts engagement and mood. However, no studies investigated the correlation between Chinese college students' arts engagement, anxiety, and resilience. There has been no discussion of the psychological moderating effects of various types of arts engagement.

This study includes a preliminary examination to assess the adaptation and anxiety symptoms of Chinese college students concerning the COVID-19 outbreak. We also assessed predictors of anxiety and resilience among college students during the lockdown. Finally, we measured the effects of various dimensions of arts engagement on anxiety and resilience.

## Methods

This survey was conducted from March 15, 2022, to April 15, 2022. At the time, there was a wave of SARS-CoV-2 infections in Shanghai, China. In order to curb the spread of the pandemic, delay return to academic institution was required for students in Shanghai. Strict management measures for students have also been carried out by colleges and universities around the country, that students are required to keep in academic institution as much as possible without activities outside.

Several academic institutions implemented strict closure measures, and arts and cultural centers across the country closed (38). The survey measured mental health indicators such as anxiety and resilience as outcome variables. We measured demographics, COVID-19 risk profiles, and frequency of receptive arts engagement in the previous year as predictors.

### Participants

Participants come from all over China to study different majors. A total of 2,453 valid questionnaires were collected, of which 1,817 (74.1%) were from female respondents. The sample included undergraduate students (*n* = 1,997, 81.4%), masters students (*n* = 403, 16.4%), and doctoral students (*n* = 63, 2.2%). The samples were from several majors, including humanities and social sciences majors (*n* = 1,544, 62.9%) and natural science majors (*n* = 909, 37.1%). Most of the 2,453 reported that they were in academic institution and in isolation (*n* = 2,119, 86.4%), and 442 were from medium-high-risk areas (18%) (for the complete data, see Table 1).

**Table 1:** Demographics.

**Variable**	**Sample,** ***n*** **(%)**
**Sex**	
Female	1,817 (74.1%)
Male	636 (25.9%)
**Provinces that participants resided**	
Hunan	865 (35.2%)
Jiangxi	573 (23.3%)
Shanghai	447 (18.2%)
Zhejiang	238 (9.7%)
Guangdong	129 (5.3%)
Other provinces	211 (8.6%)
**Learning level**	
Undergraduate	1,997 (81.4%)
Master student	403 (16.4%)
Doctoral student	53 (2.2%)
**Major**	
Natural science	909 (37.1%)
Humanities and social sciences	1,544 (62.9%)
**Living status**	
living alone	81 (3.3%)
Two people sharing	64 (2.6%)
3–4 people sharing	1,422 (58%)
Five people or more	886 (36.1%)
**Epidemic situation**	
Low risk area	2,011 (82.0%)
Medium risk area	404 (16.5%)
High risk area	38 (1.5%)
**Current living situation**	
Free activities without returning to academic institution	154 (6.3%)
Closed management without returning to academic institution	84 (3.4%)
Back to academic institution, free activities	96 (3.9%)
Back to academic institution, closed management	2,119 (86.4%)

### Procedure

We collated data using the Wenjuanxing Survey (an online data collection platform, https://www.wjx.cn/). To recruit the sample, college counselors in various universities were approached by the research team to disseminate the survey information to the target population (i.e., university students) *via* their respective online social communities, so as to assist to launch the online survey. During this period, these colleges and universities have taken relatively strict closing measures for going out. In order to ensure the universality of the research results, we distributed the questionnaire to all majors as far as possible, and the participants were composed of undergraduates, masters and doctoral students. The research ethics committee of the first author's institution approved the study before recruiting volunteers. Before collecting data, we asked recruited participants to provide informed consent. Before completing the online questionnaire independently, participants were informed that the survey was anonymous and confidential, and they could stop participating at any time.

There were two inclusion criteria for participants. These were that they were (i) students studying in an undergraduate or a post-graduate university program in mainland China, and (ii) aged 18 years or over. The invalid questionnaires were eliminated based on response time, and the abnormal questionnaires with response time lower than 90s (too fast) or longer than 600s (unreasonably long) were eliminated based on pre-test of the questionnaire completion. Then, the testers reviewed them individually to eliminate invalid questionnaires (i.e., with incomplete answers or answer in repetition). After excluding invalid questionnaires, we collected 2,453 valid questionnaires from the final 2,708 primary questionnaires for a valid response rate of 90.58%.

### Material

#### Sociodemographic variables

Participants were asked to report their gender, stage of study, major, city, and residence. They were also asked to report the outbreak and closure measures in their academic institution's area (see Supplementary Appendix 1). These variables have been shown in past research to be significantly related to students' mental health (39).

#### COVID-19-related risk status

Participants were asked to report whether they were exposed to six risk situations associated with the COVID-19 pandemic (including self-isolation and knowing someone in quarantine) (13) (see Supplementary Appendix 2). The total score range was 0–100. “I tested (once) positive for COVID-19” scored 100 points. “I am currently under mandatory quarantine or medical observation” scored 83.33 points. “I know some people who have tested (previously) positive for COVID-19” scored 66.67. “I have been required to be in mandatory quarantine or medical observation” scored 50 points. “My family or close friends are or have been in quarantine” scored 33.33. “I know someone who is or has been quarantined” scored 16.67. None of the above was counted as 0 points.

Participants were also asked to report whether there were changes in behavior associated with the pandemic (see Supplementary Appendix 2) (13). “Yes” answers were calculated as a continuous variable ranging from 0 to 11.

#### Level of arts engagement

This study measures artistic and cultural engagement through 12 items. The items were adapted from research by Mak et al. (40), who identified four potential categories of arts engagement during a lockdown: digital arts and writing, music activities, crafts, and reading activities. The Kaiser-Meyer-Olkin value was 82.4. Kaiser's criterion of eigenvalues >1 indicated a four-factor structure.

When considering art projects, we mainly focus on active arts engagement. Among the 12 items, 10 are active artistic activities and 2 are receptive artistic activities. Due to the closed policy during the epidemic period, it is difficult for students to go out to participate in receptive art activities (e.g., watching dance performances and art exhibitions). At the same time, compared with receptive arts engagement, active arts engagement mainly occurred mainly alone (41). Tymoszuk et al. (41) found that reading and listening to recorded music are the most popular and most likely to participate in art activities, so we add these two receptive activities to the list. Participants were asked to rate how often they had participated in arts activities in the previous year on a scale from 1 (not at all) to 6 (at least once a week) (see Supplementary Appendix 3).

#### COVID-19 anxiety symptoms

We used the seven-item Generalized Anxiety Disorder Scale (GAD-7) to measure anxiety levels over the previous 2 weeks. GAD-7 is one of the most widely used anxiety disorders testing and screening tools (42). In 2010, GAD-7 was translated into a Chinese version and validated in outpatients in general hospitals, α = 0.898 (43). Participants were asked to report the frequency of their anxiety symptoms over the past 2 weeks on a scale from 1 (not at all) to 4 (nearly every day). The total score ranges from 0 to 21, with a cut-off value of 10 for identifying cases of GAD; 0–4 indicates no or extreme anxiety, 5–9 is mild, 10–14 is moderate, and 15–21 is severe depression.

#### COVID-19 resilience

We used the Connor-Davidson Resilience Scale to measure resilience levels (44), defined as the ability to thrive in adversity. Higher scores reflect resilience. We used a simplified version of the scale, including 10 items, α = 0.83 (45). Participants were asked to report how the pandemic affected their resilience on a scale from 1 (not true at all) to 5 (correct almost all the time).

### Data analysis

Pearson correlations were calculated to establish a preliminary link between study variables (means, standard deviations, and correlations of study variables). We then performed a multilevel linear regression analysis, using anxiety as the dependent variable. In the independent variables column, we entered demographic variables and behavioral changes caused by COVID-19 in step 1. We entered the frequency of accepting arts participation, exposure to COVID-19, and resilience in step 2. We then calculated the interaction of frequency of receiving art participation, exposure to COVID-19-related conditions, and resilience to anxiety.

We then used resilience as the dependent variable. In the column of independent variables, demographic variables and behavioral changes caused by the new crown were entered into step 1. We entered the frequency of art participation and exposure to COVID-19 in step 2. We then calculated the interaction of frequency of receiving art participation and exposure to COVID-19-related conditions on resilience.

Finally, we measured the relationship between each dimension of art participation and anxiety and resilience. We used the scores of art participation in each dimension as independent variables and anxiety and resilience as dependent variables.

## Results

### Anxiety levels of college students

Among 2,453 college students, 56.3% had no anxiety symptoms. The proportions of students with mild, moderate, moderate to severe and severe anxiety were 29.8, 6.0, 5.3, and 2.6%, respectively.

### Correlation analysis between variables

Table 2 displays the mean, standard deviation, and correlation among the primary variables in the study. All major variables were significantly correlated; however, the correlation between variables was low to moderate (46). Specifically, exposure to COVID-19 was positively correlated with COVID-19 anxiety (*r* = 0.123, *p* < 0.01) and negatively correlated with resilience (*r* = −0.085, *p* < 0.01). Resilience was negatively correlated with COVID-19 anxiety in college students (*r* = −0.348, *p* < 0.01). The frequency of receiving art participation was negatively correlated with anxiety levels of COVID-19 (*r* = −0.119, *p* < 0.01) and positively correlated with resilience levels (*r* = 0.143, *p* < 0.01).

**Table 2:** Demographics and correlations table for the study variables.

	**Mean**	**SD**	**1**	**2**	**3**	**4**	**5**	**6**	**7**	**8**	**9**	**10**	**11**
1. Sex^a^	—	0.438	—										
2. Learning level^b^	—	0.436	−0.002	—									
3. Major^c^	—	2.926	0.251^**^	−0.034	—								
4. Living conditions^d^	—	0.669	−0.108^**^	−0.300^**^	−0.134^**^	—							
5. Epidemic situation^e^	—	0.434	−0.070^**^	0.067^**^	−0.027	0.104^**^	—						
6. Current living situation^f^	—	0.809	−0.016	−0.123^**^	0.002	0.336^**^	0.000	—					
7. GAD	4.72	4.892	−0.020	−0.003	0.036	0.024	0.049^*^	−0.065^**^	—				
8. Resilience	26.44	7.758	−0.069^**^	0.105^**^	−0.014	−0.041^*^	−0.009	0.010	−0.348^**^	—			
9. ArtE	21.34	11.772	0.103^**^	−0.040^*^	0.216^**^	−0.044^*^	0.007	0.000	−0.119^**^	0.143^**^	—		
10. Behavioral change	9.47	2.215	0.106^**^	0.001	0.044^*^	−0.011	−0.006	−0.005	−0.004	0.047^*^	0.029	—	
11. Exposure to COVID-19–related situations	12.46	22.909	0.019	0.097^**^	0.012	0.006	0.241^**^	−0.009	0.123^**^	−0.085^**^	0.070^**^	−0.038	—

Total N = 2,453.

ArtE indicates frequency of attending arts-based events; GAD, general anxiety disorder; Resilience, the Connor-Davidson Resilience Scale.

a Sex: male was coded as “1” and female as “2.”

b Learning level: undergraduate was coded as “1,” post-graduate as “2” and doctoral candidate as “3.”

c Major: natural science was coded as “1” and humanities and social sciences as “2.”

d Living conditions: living alone was coded as “1,” two people sharing as “2,” 3–4 people sharing as “3” and five people or more as “4.”

e Epidemic situation: low risk area was coded as “1,” medium risk area as “2” and high risk area as “3.”

f Current living situation: free activities without returning to school as “1,” Closed management without returning to school as “2” Closed management without returning to school as “3” and back to school, closed management as “4.”

** Correlation is significant at the 0.01 level (two-tailed).

* Correlation is significant at the 0.05 level (two-tailed).

The resilience of college students was correlated with gender (*r* = −0.69, *p* < 0.01), women showed a lower level of resilience. And the resilience was positively correlated with the learning stage of college students (*r* = 0.105, *p* < 0.01), graduate students reported higher resilience than undergraduates. The degree of art participation correlated with gender (*r* = 0.105, *p* < 0.01). The behavioral changes caused by COVID-19 were correlated with gender (*r* = 0.106, *p* < 0.01). The degree of art participation was correlated with professional type (*r* = 0.216, *p* < 0.01).

### Hierarchical multiple linear regression for predicting anxiety symptoms

Hierarchical regression analysis for predicting anxiety showed that college students who had returned to academic institution and were under lockdown reported lower anxiety symptoms (β = −0.83, *t* = −3.883, *p* < 0.001). Academic institutions located in higher-risk areas reported higher levels of anxiety symptoms (β = 0.481, *t* = 2.097, *p* < 0.05) (Table 3).

**Table 3:** Hierarchical multiple linear regression for predicting anxiety symptoms.

**Predictor**	***R***	***B***	**SE**	**β**	***t***	***p*** **-Value**
**Step 1**						
Sex	0.105^***^	−0.284	0.234	−0.025	−1.215	0.224
Learning level		0.016	0.228	0.001	0.068	0.946
Major		0.515	0.213	0.051	2.422	0.016^*^
Living conditions		0.377	0.166	0.052	2.264	0.024^*^
Epidemic situation		0.481	0.230	0.043	2.097	0.036^*^
Current living situation		−0.503	0.129	−0.083	−3.883	0.000^***^
Behavioral change		−0.006	0.045	−0.003	−0.135	0.893
**Step 2**						
Resilience	0.369^***^	−0.207	0.012	−0.328	−17.226	0.000^***^
ArtE		−0.033	0.008	−0.079	−4.123	0.000^***^
Exposure to COVID-19–related situations		0.021	0.004	0.100	5.310	0.000^***^
Total	0.383^***^					

Dependent variable: GAD.

Total N = 2,453.

ArtE indicates frequency of attending arts-based events; GAD, general anxiety disorder; Resilience, the Connor-Davidson Resilience Scale.

* p < 0.05.

*** p < 0.001.

In the second step input, students with greater exposure to COVID-19 reported higher levels of anxiety symptoms (β = 0.021, *t* = 5.310, *p* < 0.001). Lower resilience was associated with higher anxiety symptoms (β = −0.207, *t* = −17.226, *p* < 0.001). Individuals who participated in art activities less often reported higher symptoms of anxiety (β = −0.033, *t* = 5.310, *p* < 0.001). These variables significantly explained 38.3% of the variance in anxiety symptoms among college students.

To calculate the interaction effect of main variables on anxiety, college students were grouped according to exposure to COVID-19, art participation, and resilience (see Table 4).

**Table 4:** Between-subjects factors.

		**Value label**	***N***
Resilience	1	≤ 19	327
	2	20–26	819
	3	27–33	922
	4	34+	385
ArtE	1	≤ 22	1,472
	2	23+	981
Exposure to COVID-19–related situations	1	≤ 12	1,653
	2	12–25	344
	3	25–55	270
	4	55+	186

The main effect of the frequency of art participation was significant [*F* (1) = 4.657, *p* = 0.031, *p* < 0.05], indicating that the level of art participation has a significant impact on anxiety scores (Table 5). The main effect of resilience was significant [*F* (1) = 35.025, *p* = 0.00, *p* < 0.01], indicating that the resilience of college students has a significant impact on anxiety scores. The main effect of exposure to COVID-19 was significant [*F* (1) = 7.888, *p* = 0.00, *p* < 0.01], indicating that exposure to COVID-19 had a significant impact on anxiety scores. The interaction effect of these three factors was significant [*F* (9) = 2.669, *p* = 0.004, *p* < 0.01], indicating that the level of arts engagements, resilience, and exposure to COVID-19 had a significant interaction effect on college students' anxiety.

**Table 5:** Tests of between-subjects effects.

**Source**	**Type III sum of squares**	**df**	**Mean square**	***F***	**Sig**.
ArtE	97.439	1	97.439	4.657	0.031
Resilience	2,198.641	3	732.880	35.025	0.000
Exposure to COVID-19–related situations	495.138	3	165.046	7.888	0.000
ArtE * resilience	88.932	3	29.644	1.417	0.236
ArtE * exposure to COVID-19–related situations	88.161	3	29.387	1.404	0.240
Resilience * exposure to COVID-19–related situations	164.311	9	18.257	0.873	0.549
ArtE * resilience * exposure to COVID-19–related situations	502.630	9	55.848	2.669	0.004

Dependent variable: GAD.

R Squared = 0.137 (Adjusted R Squared = 0.126).

### Hierarchical multiple linear regression for predicting resilience symptoms

We performed hierarchical regression analysis to predict resilience (see Table 6). Boys reported higher resilience (β = −0.079, *t* = −3.791, *p* < 0.001). College students with higher learning stages reported higher resilience (β = 0.099, *t* = 4.654, *p* < 0.001). College students who experienced more behavioral changes during COVID-19 reported higher resilience (β = 0.055, *t* = 2.719, *p* < 0.01). College students who received art participation more frequently also reported higher resilience (β = 0.099, *t* = 7.521, *p* < 0.001). Students with less exposure to COVID-19 reported higher levels of resilience (β = −0.032, *t* = −4.802, *p* < 0.001). These variables significantly predicted 23% of college students' variance in resilience symptoms.

**Table 6:** Hierarchical multiple linear regression for predicting resilience.

**Predictor**	***R***	***B***	**SE**	**β**	***t***	***p*** **-Value**
**Step 1**						
Sex	0.144^***^	−1.401	0.369	−0.079	−3.791	0.000^***^
Learning level		1.690	0.363	0.099	4.654	0.000^***^
Major		0.193	0.373	0.012	0.517	0.605
Living conditions		−0.324	0.263	−0.028	−1.233	0.218
Epidemic situation		−0.332	0.362	−0.019	−0.917	0.359
Current living situation		0.296	0.204	0.031	1.450	0.147
Behavioral change		0.192	0.070	0.055	2.719	0.007^**^
**Step 2**						
ArtE	0.172^***^	0.099	0.013	0.150	7.521	0.000^***^
Exposure to COVID-19–related situations		−0.032	0.007	−0.096	−4.802	0.000^***^
Total	0.230^***^					

Dependent variable: Resilience.

Total N = 2,453.

ArtE indicates frequency of attending arts-based events; GAD, general anxiety disorder; Resilience, the Connor-Davidson Resilience Scale.

** p < 0.01.

*** p < 0.001.

To calculate the interaction of the main variables on resilience, college students were grouped according to exposure to COVID-19, art participation, and resilience (see Table 4). The statistical results of the data in Table 7 show that the primary effect of the frequency of art participation is significant [*F* (1) = 30.444, *p* = 0.00, *p* < 0.01], suggesting that the level of art participation has a significant impact on resilience. The primary effect of exposure to COVID-19 was significant [*F* (3) = 7.401, *p* = 0.00, *p* < 0.01], suggesting that exposure to COVID-19 has a significant impact on resilience. The interaction effect of these two factors was significant [*F* (3) = 2.669, *p* = 0.047, *p* < 0.05], suggesting that the level of arts engagements and exposure to COVID-19 had a significant interaction effect on the anxiety level of college students.

**Table 7:** Tests of between-subjects effects.

**Source**	**Type III sum of squares**	**df**	**Mean square**	***F***	**Sig**.
ArtE	1,797.869	1	1,797.869	30.444	0.000
Exposure to COVID-19–related situations	1,311.253	3	437.084	7.401	0.000
ArtE * Exposure to COVID-19–related situations	469.548	3	156.516	2.650	0.047

Dependent variable: Resilience.

R Squared = 0.021 (Adjusted R Squared = 0.018).

### Effects of dimensions of art participation on anxiety and resilience

We used the scores of art participation in each dimension as independent variables and conducted multiple linear regression analyzes with anxiety and resilience as dependent variables (Table 8). We performed multiple linear regression analyzes with the scores of art participation in each dimension as the independent variable and anxiety as the dependent variable. The regression model has significant statistical significance (*F* = 21.366, *p* < 0.001). The independent variable can explain 18.4% of the changes in the subjects' anxiety scores, which has a certain degree of explanation. The significance test results showed that the effects of music activity and reading on anxiety scores were statistically significant (*p* < 0.001).

**Table 8:** Hierarchical multiple linear regression for predicting anxiety symptoms.

**Dimension of art participation**	***R***	***F***	***B***	**SE**	**β**	***t***	***p*** **-Value**
**Dependent variable: GAD**							
**Total**							
Digital art and writing	0.184^***^	21.366	0.088	0.059	0.037	1.484	0.138
Music activities			−0.327	0.059	−0.129	−5.561	0.000^***^
Handicrafts			0.047	0.067	0.017	0.698	0.485
Reading activities			−0.180	0.031	−0.123	−5.749	0.000^***^
**Dependent variable: Resilience**							
**Total**							
Digital art and writing	0.165^***^	17.075	0.259	0.095	0.068	2.744	0.006^**^
Music activities			0.281	0.094	0.070	3.006	0.003^**^
Handicrafts			−0.070	0.107	−0.016	−0.651	0.515
Reading activities			0.210	0.050	0.090	4.201	0.000^**^

Total N = 2,453.

** p < 0.01.

*** p < 0.001.

The four dimensions of art participation are independent variables, and resilience is the dependent variable for multiple linear regression analysis. The results are shown in Table 8, and the regression model has significant statistical significance (*F* = 17.075, *p* < 0.001). The independent variable can explain 16.5% of the change in the subjects' resilience score, which has a certain degree of explanation. The results of the significance test showed that digital art, music activity, and reading had a statistically significant effect on the resilience score (*p* < 0.01).

## Discussion

Public health emergencies have many impacts on college students, which can manifest in anxiety, fear, and worry. At the same time, resilience as a psychological resource can help college students fight the epidemic's negative emotions (33). The purpose of this study was to investigate the factors affecting anxiety and resilience. At the same time, we studied the relationship between arts engagement and college students' anxiety and resilience during COVID-19.

### Predictors of anxiety symptoms

The growing number of patients and suspected cases has raised public concerns about being infected in this outbreak, which has increased anxiety (47). The results of this study show that, in this round of the pandemic, 43.7% of college students suffered from varying degrees of anxiety, of which 2.6% showed severe anxiety. The epidemic might impact college students' study and future employment, increasing anxiety (48). Anxiety might also be caused by social distancing. Anxiety disorders are more likely to develop and worsen without interpersonal communication (49).

The results of the correlation analysis showed that the anxiety generated in COVID-19 was not significantly different in terms of gender. This finding is similar to Cao et al. (7) and suggests that male and female university students experience similar stress and negative emotions due to the pandemic.

There was no significant difference in anxiety levels among students at different learning stages, which was different from the results of Wang et al. (48). These authors investigated the anxiety levels of college students during home distance learning at the beginning of the epidemic and found that undergraduates were more affected than graduate and doctoral students. Most students in the present study had already returned to academic institution, so distance learning was not complex.

Multiple linear regression analysis showed that students with greater exposure to COVID-19 reported higher anxiety symptoms. This finding is similar to Wang et al. (50) and Tan et al. (51). In addition, students located in higher-risk areas reported higher levels of anxiety symptoms. This finding is consistent with studies conducted in Chengdu and Chongqing, China (52). The COVID-19 pandemic may significantly impact the risk of anxiety symptoms among Chinese college students. This finding may be due to the highly contagious characteristics of COVID-19 pneumonia (53) This finding may be due to the highly contagious characteristics of COVID-19 pneumonia. As the number of new confirmed cases rises, measures to limit the movement of people (e.g., lockdowns) are required to control the pandemic. However, measures restricting freedom may increase mental distress (54).

College students who had not returned to academic institution reported higher levels of anxiety symptoms. This finding might be because not returning to academic institution means remote learning, which could impact academic achievement. Cao et al. (7) found the impact on academics to be a stressor for COVID-19. Additionally, students may experience a lack of social support once off campus or may experience stigma associated with seeking mental health care. This situation can hinder their ability to seek help when they develop symptoms in events such as a pandemic (55).

Individuals who received less frequent arts engagement also reported higher symptoms of anxiety (β = −0.033, *t* = 5.310, *p* < 0.001). This finding is consistent with the findings of Keisari et al. (13) and suggests that arts engagement appears to be a coping resource and risk-reducing factor for developing mental health problems in college students. Artistic activity involves imagination, sensory activation, cognitive stimulation, and social interaction. These activities can facilitate psychological, physical, social, and behavioral responses related to the management of mental health and wellbeing (10). Art can support individual coping by helping individuals avoid stressors (e.g., by providing distractions) and reassessing problems they may be facing (e.g., by providing time and space to resolve questions). Art can help students self-regulate their emotions and boost their self-confidence to face challenges better (56).

Higher resilience was associated with lower anxiety symptoms. This finding is consistent with Savitsky et al. (57) and Tan et al. (51), suggesting that resilience has a powerful effect on mental health during a pandemic. Individuals with higher resilience reduce their negative emotions by actively participating in society to achieve higher levels of optimism (58). Individuals with high psychological resilience clearly understand the meaning of positive coping styles and overcome the impact of negative emotions on themselves during times of adversity (59). Individuals with positive coping styles have better mental health than those with negative coping styles (60). Therefore, people who are highly resilient to public health emergencies are less likely to experience negative emotional symptoms. Resilience protects the mental health of refugees and natural disaster survivors (61).

Arts engagement, resilience, and exposure to COVID-19 had a significant interaction effect on the anxiety level of college students. This finding is consistent with the results of Keisari et al. (13), who showed that older adults showed a significant interaction between resilience and receptive arts engagement frequency during COVID-19. Participants with higher levels of participation in the arts or those with higher levels of resilience reported lower levels of COVID-19 anxiety. Only those with low resilience and low engagement in receiving art showed very high anxiety levels. This finding suggests that participation appears to be a coping resource and risk-reducing factor for developing mental health problems in college students (62).

### Predictors of resilience

Boys reported higher resilience than girls. This finding is similar to the findings of Afshari et al. (63) and Bozdag et al. (36), suggesting differences in physiological structure and function between males and females who exhibit different neurobiological responses to stressors (64).

Graduate students reported higher resilience than undergraduates. Graduate students are older and have more experience than undergraduates. Resilience is a dynamic acquisition or skill set process that increases with age (65), suggesting that flexibility improves. Graduate students have more knowledge and abilities than undergraduates. Schwind et al. (66) found higher resilience scores among literate earthquake survivors. This finding suggests that a stronger intellectual background improves understanding of various situations; these individuals might acquire or access information resources to adapt to emergencies or disasters, reducing their vulnerability.

College students who experienced more behavioral changes during COVID-19 reported higher resilience. Vannini et al. (67) also found that resilience positively correlated with COVID-19 coping strategies. Individuals with high resilience understand the meaning of positive coping styles and overcome the impact of negative emotions in adversity (59). Behaviors such as reducing handshakes and physical contact are positive responses to the epidemic. In addition, resilience may be related to the sense of control. It refers to the extent to which individuals feel they can influence events or situations in their own life. The sense of control can serve as a potential buffer to prevent a sharp decline in happiness (68). Sense of control is also related to wellbeing and other pandemic protecting behaviors such as getting vaccinated (69). Therefore, college students with stronger sense of control may show more behavioral changes. Students with greater exposure to COVID-19 reported lower levels of resilience. This finding is similar to Killgore et al. (70), who found that psychological resilience decreased significantly during the first few weeks of the COVID-19 lockdown in the United States. These findings suggest that the environment might affect college students' psychological resilience.

College students who received a higher frequency of arts engagement reported higher resilience, consistent with the findings of Diamond et al. (71). There is a positive correlation between creativity and resilience (72), and high arts engagement may enhance individual creativity, suggesting that high arts engagement may enhance resilience. During the pandemic, arts engagement appears to enhance the pro social behavior of college students (73), and students can experience more social support. As their perceived social support increases, so does resilience (74).

The interaction effect analysis suggests that the degree of accepting arts engagement and exposure to COVID-19 has a significant interaction effect on the resilience of college students, suggesting that arts engagement can neutralize the impact of the external environment on the mental health of college students. This result is similar to that of Tubadji et al. (73) and suggests that creativity is therapeutic for victims of natural or manufactured disasters and promotes post-traumatic growth (75). High arts engagement during a pandemic can foster creativity in college students to a certain extent, thereby enhancing resilience.

### The role of different artistic dimensions on resilience and anxiety

Our findings suggest that arts engagement is related to the level of anxiety and resilience of college students during the epidemic. A multiple linear regression analysis was performed to study the effects of different dimensions of arts engagement on anxiety and resilience. Among the four dimensions of arts engagement, musical activity and reading had statistically significant effects on anxiety scores (*p* < 0.001). Digital arts, musical activity, and reading had a statistically significant (*p* < 0.01) effect on resilience scores.

Students with high frequency of musical activity reported lower anxiety levels. Students with a high frequency of participation in music activities also reported higher levels of resilience. Participating in musical activities may contribute to emotion regulation in college students, similar to Chen et al. (76) and Bu et al. (12). Pleasing music activates brain structures and stimulates the release of dopamine, serotonin, and endorphins, positive neurotransmitters that induce euphoria, pleasure, and relaxation (77). Music distracts students from anxiety-inducing stimuli (77). During a lockdown, social isolation, economic instability, the challenges of distance learning, and the fear of being infected can all stimulate anxiety in college students, and music can distract students from these stimuli. There is an association between music activity and low levels of anxiety.

Students with high frequency of participation in reading activities reported lower anxiety levels and higher resilience, suggesting that reading activities may serve as a resource to maintain the mental health of college students during isolation. This finding is consistent with previous studies (12, 78, 79). Books can provide an immersive simulation of the real social world, where readers can project themselves into situations and enhance their understanding of characters (80). In the process, readers can activate past personal memories associated with the narrative environment. This activity can help readers comprehend personal experiences (81), helping readers to ease negative emotions. Research shows that reading for just 6 min a day is an effective strategy for reducing stress levels by up to 68%, enabling people to clear their minds and reduce physical tension (79). The environment less restricts reading, and reading may be an important recreational tool to give psychological support to college students under social isolation conditions.

Students with a high frequency of participation in digital arts and writing reported higher resilience; however, there was no significant difference in anxiety levels. The digital art and writing categories include artistic activities such as photography, creating digital artwork, making videos, and writing (i.e., creative activities). Engaging in digital art and writing can promote self-development and improve the ability to cope with crises, as found by Mak et al. (40). This activity requires participants to be creative, and there is a positive correlation between creativity and resilience (72). These creative activities can provide opportunities to capture past life events. Documenting positive events and reflecting on happy memories are standard techniques for improving overall mood and wellbeing (82). Students more engaged in digital arts and writing activities reported resilience. Nevertheless, the frequency of participation in digital art and writing did not affect anxiety during the pandemic. This finding may be explained by the difficulty in carrying out activities such as photography and filming due to the venue's impact during the lockdown.

### Strategies for relieving college students' negative emotions toward COVID-19

This study investigated the predictors of college students' anxiety and resilience during the pandemic, especially the moderating effect of arts engagement. In view of the research results, some suggestions were proposed to assist college students in stressful situations (such as infectious disease outbreaks or natural disasters) to cope with negative emotions.

First, it is found in this study that 43.7% of the enrolled college students have suffered from different degrees of anxiety, among which 2.6% indicated serious anxiety. Therefore, the university administrative departments should develop effective screening procedures, pay close attention to students' exposure to stressors and mental health status. For students who are plagued by psychological problems, the relevant department as well as student instructors should provide prompt and effective intervention.

Second, this study showed that arts engagement is negatively correlated to the level of negative emotions such as anxiety, which can be used as an important resource for college students to cope with stress during the outbreak of the pandemic. The pandemic may result in the restriction of access to public spaces, galleries, exhibitions, museums and art venues, greatly affecting the development of receptive art activities (37). As to the selection of artistic activities, it was revealed that participatory artistic activities such as singing, painting and writing can help to alleviate students' negative emotions. The artistic materials required for these activities are accessible (e.g., colored pencils, books, and sketchbooks) regardless of economic conditions. Therefore, universities and educational administrative departments should develop more participatory artistic activities for students. Simultaneously, online artistic activities can be taken as a parallel option to alleviate students' anxiety during covid-19. For example, increasing number of art museums provide online art participation for exploring digital virtual platforms for the public (83). In this way, students can make sufficient use of network resources to enrich opportunities to experience art and culture. Policy developers should consider providing online art resources for college students during the lockdown by using online technology and other platforms.

Third, this study indicated the association of higher resilience with lower anxiety symptoms. Elevated individual resilience has been proved as an essential strategy to help individuals rebound from adversity in the face of various stressors and stress-induced events and trauma (84). Therefore, schools should actively try relevant strategies to improve students' resilience and cultivate their positive attitudes in daily life. The positive coping style can contribute to coping with the issues that may occur in the COVID-19, reducing the pressure to prevent depression. During COVID-19 the academic institution can also encourage students to consolidate useful routines (restore the regularity and normality of daily life) and develop new and meaningful routines (for example, develop new interests). These measures can help students enhance or develop the capability to adapt to change.

## Limitations and future work

This study investigated the predictors of college students' anxiety and resilience during the pandemic, especially the moderating effect of art participation, and compared the moderating effects of different dimensions of arts engagement.

There are some limitations of this study. First, given the limited resources and the time-sensitive COVID-19 outbreak, we adopted a snowball sampling strategy. The snowball sampling strategy is not based on a random selection of samples and cannot represent the samples' overall distribution. This method may result in a possible bias in the gender distribution of the sample. For example, women had lower resilience, which could be attributed to the slightly higher proportion of female respondents. Second, this study is cross-sectional, and the lack of comparisons not affected by the pandemic is a point-in-time comparison; therefore, causality cannot be established. Third, to document various forms of art engagement during the lockdown, we did not specify whether respondents participated in person or virtually; future work needs to explore the impact of online art engagement on emotion regulation. Finally, the study did not examine the long-term effects of artistic participation, which may be the subject of future research.

During COVID-19, strict quarantine measures have maintained the public's physical health but put their mental health at risk. While college students may be at lower risk for serious complications associated with COVID-19, they face significant disruption following school closures and social distancing measures. This study shows that arts participation appears to be a coping resource and risk-reducing factor for developing mental health problems in college students. Policymakers should consider using online technology and other platforms to help college students during the lockdown. Examples include digital art events in online groups, online tours in museums and galleries, and movies and videos of concerts and performances during social distancing.

## Data availability statement

The raw data supporting the conclusions of this article will be made available by the authors, without undue reservation.

## Ethics statement

The study was approved by the ethical review board of Central South University before data collection. All methods were carried out in accordance with local guidelines and regulations. The written informed consent was obtained from each participant.

## Author contributions

YC: supervision, conception and design of the study, revising the article for important intellectual content, and final approval of the version to be submitted. XZ: conception and design of the study, analysis and interpretation of data, and drafting the article. LT, JC, and YW: revising the article for important intellectual content. All authors contributed to the article and approved the submitted version.

## Funding

This work was supported by Humanities and Social Sciences Research Project of the Ministry of Education (Grant Number: 20YJCZH012), The Second Batch of New Engineering Research and Practice Projects (Grant Number: E-YGJH20202805), Educational Science Planning Project of Hunan Province (Grant Number: XJK21CGD004), Research Project on Educational Teaching Reform of Central South University (Grant Number: 2021jy094), Post-graduate Scientific Research Innovation Project of Hunan Province (Grant Number: CX20210141), and Independent Exploration and Innovation Project for Graduate Students of Central South University (Grant Number: 2021zzts0464).

## Conflict of interest

The authors declare that the research was conducted in the absence of any commercial or financial relationships that could be construed as a potential conflict of interest.

## Publisher's note

All claims expressed in this article are solely those of the authors and do not necessarily represent those of their affiliated organizations, or those of the publisher, the editors and the reviewers. Any product that may be evaluated in this article, or claim that may be made by its manufacturer, is not guaranteed or endorsed by the publisher.
