# Supplementary material for: The influence of arts engagement on the mental health of isolated college students during the COVID-19 outbreak in China
Source: Front Public Health. 2022 Nov 15;10:1021642. doi: 10.3389/fpubh.2022.1021642 (PMC9706106; doi:10.3389/fpubh.2022.1021642)
Supplement: Supplementary file 1 [file Data_Sheet_1.docx]

Appendix 1. Sociodemographic Questionnaire

| **Questions** | | | **Options** | | |
| --- | --- | --- | --- | --- | --- |
| 1. Your gender? |  |  | | Male | Female |
| 2. What stage of study are you in? |  | Undergraduate | | Postgraduate | Doctoral candidate |
| 3. Which of the following categories does your major belong to? |  |  | | Natural Science | Humanities and Social Sciences |
| 4. What is your current living status? | Living alone | Two people sharing | | 3-4 people sharing | Five people or more |
| 5. The risk profile of your school district |  | Low risk area | | Medium risk area | High risk area |
| 6. What is your current living situation? | Free activities without returning to school | Closed management without returning to school | | Back to school free activities | Back to school, closed management |

Appendix 2. COVID-19 related risk status

| **Questions** | **Options** | |
| --- | --- | --- |
| **Which of the following statements about the COVID-19 pandemic applies to your current situation** | | |
| I am currently under mandatory quarantine or medical observation | Yes | No |
| I have been required to be in mandatory quarantine or medical observation | Yes | No |
| I tested (once) positive for COVID-19 | Yes | No |
| I know someone who is or has been quarantined | Yes | No |
| My family or close friends are or have been in quarantine | Yes | No |
| I know some people who have tested (previously) positive for COVID-19 | Yes | No |
| **How has your behavior changed since the COVID-19 outbreak?** |  |  |
| less handshake | Yes | No |
| less hugs | Yes | No |
| maintain more physical distance from others | Yes | No |
| less social | Yes | No |
| Reduce the number of trips | Yes | No |
| Reduce the number of times you meet with friends | Yes | No |
| Use a mask or gloves | Yes | No |
| Reduce the number of trips to public places | Yes | No |
| Wash your hands frequently and stay clean | Yes | No |
| Stock up in response to a crisis - gloves, masks, water, food, etc. | Yes | No |
| Cancellation or changes to major plans such as flights, trips, family events, etc. | Yes | No |

Appendix 3. Level of arts engagement

| **Questions** | | | | | **Options** | | |
| --- | --- | --- | --- | --- | --- | --- | --- |
| Please fill in according to the actual situation, the frequency of your participation in the following activities in the past year | | | | | | | |
| Singing | almost everyday | Once or twice a week | Once or twice a month | every few months | | Once or twice a year | not at al |
| Playing a musical instrument | almost everyday | Once or twice a week | Once or twice a month | every few months | | Once or twice a year | not at al |
| Painting, drawing, printmaking or sculpture | almost everyday | Once or twice a week | Once or twice a month | every few months | | Once or twice a year | not at al |
| Reading books, stories or poetry | almost everyday | Once or twice a week | Once or twice a month | every few months | | Once or twice a year | not at al |
| Textile crafts, e.g., embroidery, crocheting or knitting | almost everyday | Once or twice a week | Once or twice a month | every few months | | Once or twice a year | not at al |
| Wood crafts, e.g., carving or furniture making | almost everyday | Once or twice a week | Once or twice a month | every few months | | Once or twice a year | not at al |
| Other crafts, e.g., pottery, calligraphy or jewelry making | almost everyday | Once or twice a week | Once or twice a month | every few months | | Once or twice a year | not at al |
| Creative writing | almost everyday | Once or twice a week | Once or twice a month | every few months | | Once or twice a year | not at al |
| Dancing | almost everyday | Once or twice a week | Once or twice a month | every few months | | Once or twice a year | not at al |
| Photography | almost everyday | Once or twice a week | Once or twice a month | every few months | | Once or twice a year | not at al |
| Create digital artwork or animation | almost everyday | Once or twice a week | Once or twice a month | every few months | | Once or twice a year | not at al |
| Listening to music | almost everyday | Once or twice a week | Once or twice a month | every few months | | Once or twice a year | not at al |
